# YKL-40/c-Met Expression in Rectal Cancer Biopsies Predicts Tumor Regression following Neoadjuvant Chemoradiotherapy: A Multi-Institutional Study

**DOI:** 10.1371/journal.pone.0123759

**Published:** 2015-04-15

**Authors:** Rebecca Senetta, Eleonora Duregon, Cristina Sonetto, Rossella Spadi, Massimiliano Mistrangelo, Patrizia Racca, Luigi Chiusa, Fernando H. Munoz, Umberto Ricardi, Alberto Arezzo, Adele Cassenti, Isabella Castellano, Mauro Papotti, Mario Morino, Mauro Risio, Paola Cassoni

**Affiliations:** 1 Department of Medical Sciences, University of Turin, Turin, Italy; 2 Department of Oncology, University of Turin, Turin, Italy; 3 SSCVD Colorectal Cancer Unit, City of Health and Science Hospital of Turin, Turin, Italy; 4 Digestive and Colorectal Surgery, Centre of Minimal Invasive Surgery, University of Turin, Turin, Italy; 5 Candiolo Cancer Institute—FPO (Fondazione del Piemonte per l'0ncologia), IRCCS (Istituto di Ricovero e Cura a Carattere Scientifico), Candiolo, Turin, Italy; University of Florida, UNITED STATES

## Abstract

**Background:**

Neoadjuvant chemo-radiotherapy (CRT) followed by surgical resection is the standard treatment for locally advanced rectal cancer, although complete tumor pathological regression is achieved in only up to 30% of cases. A clinicopathological and molecular predictive stratification of patients with advanced rectal cancer is still lacking. Here, c-Met and YKL-40 have been studied as putative predictors of CRT response in rectal cancer, due to their reported involvement in chemoradioresistance in various solid tumors.

**Material and Methods:**

A multicentric study was designed to assess the role of c-Met and YKL-40 expression in predicting chemoradioresistance and to correlate clinical and pathological features with CRT response. Immunohistochemistry and fluorescent in situ hybridization for c-Met were performed on 81 rectal cancer biopsies from patients with locally advanced rectal adenocarcinoma. All patients underwent standard (50.4 gy in 28 fractions + concurrent capecitabine 825 mg/m^2^) neoadjuvant CRT or the XELOXART protocol. CRT response was documented on surgical resection specimens and recorded as tumor regression grade (TRG) according to the Mandard criteria.

**Results:**

A significant correlation between c-Met and YKL-40 expression was observed (R = 0.43). The expressions of c-Met and YKL-40 were both significantly associated with a lack of complete response (86% and 87% of c-Met and YKL-40 positive cases, p< 0.01 and p = 0.006, respectively). Thirty of the 32 biopsies co-expressing both markers had partial or absent tumor response (TRG 2-5), strengthening their positive predictive value (94%). The exclusive predictive role of YKL-40 and c-Met was confirmed using a multivariate analysis (p = 0.004 and p = 0.007 for YKL-40 and c-Met, respectively). TRG was the sole morphological parameter associated with poor outcome.

**Conclusion:**

c-Met and YKL-40 expression is a reliable predictor of partial/absent response to neoadjuvant CRT in rectal cancer. Targeted therapy protocols could take advantage of prior evaluations of c-MET and YKL-40 expression levels to increase therapeutic efficacy.

## Background

Neoadjuvant chemoradiotherapy (CRT) followed by surgical resection according to the principle of total mesorectal excision is the standard treatment for locally advanced mid or low rectal cancer, i.e., T3-T4 lesions and/or the presence of suspected regional metastatic lymph nodes [1–4]. This approach, which allows high rates of tumor resectability and sphincter-saving procedures [5], determines tumor downstaging in 28–62%[5,6] of such cases and improves local control[7]. However, irrespective of the tumor stage, treatment response is very heterogeneous, as complete tumor pathological regression is achieved in 10–30% of cases[6,8] and many rectal cancer patients are resistant to preoperative CRT[9]. Therefore, predicting neoadjuvant CRT response may allow a more rational selection of patients who will most likely benefit from this therapy. Over the last two decades, many clinical, radiological and morphological[10–13] parameters, as well as molecular markers [14–21], have been investigated. Unfortunately, a panel of markers or a gene classifier that is useful in predicting neoadjuvant CRT response has not yet been established, and an extensive clinical validation is still needed [18,22–27]. Among possible markers, the glycoprotein YKL-40 [28] and the oncogene c-Met receptor tyrosine kinase are prognostic and chemo- and radioresistance-related markers [29–34] and are candidates for targeted therapy [28,29,31,32,35–42]. Furthermore, c-Met inhibitors were proven to increase tumor cell radiosensitivity, enhancing the ability of radiation to inhibit tumor growth and invasiveness in tumor xenografts and *in vitro* [30,37,40]. Similarly, the combination of radiotherapy and neutralizing YKL-40 antibodies showed a synergistic effect in inhibiting tumor vascularization and the progression in *in vivo* models of glioblastoma[34]. However, their role as predictive markers has not yet been tested in rectal cancer. Therefore, a multicentric study aimed at assessing the role of c-Met and YKL-40 in predicting chemo- and radioresistance was designed for 81 patients with locally advanced rectal adenocarcinoma.

Here, we show that a) c-Met and YKL-40 are independent predictors of poor response to CRT and b) tumor regression grade (TRG), but neither c-Met nor YKL-40, is the sole prognostic marker of shorter overall and disease survival.

## Materials and Methods

### Cases collection

Eighty-one rectal cancer pre-treatment endoscopic biopsies and their matched surgical specimens were retrieved from consecutive cohorts from the archives of the pathology divisions of three Italian Institutions between January 2006 and December 2012: University of Torino at Città della Salute e della Scienza (Molinette) Hospital of Turin (46 cases); San Luigi Hospital of Orbassano (16 cases); and Institute for Cancer Research and Treatment of Candiolo (19 cases). All patients had a locally advanced rectal adenocarcinoma eligible for neoadjuvant CRT (cT3-T4 stage). Fifty-one patients (63%) received CRT according to a standard capecitabine regimen (50.4 Gy in 28 fractions + concurrent capecitabine 825 mg/m^2^), whereas the remaining 30 patients (37%) underwent a XELOXART trial (50,4 Gy in 28 fractions + capecitabine 825 mg/m^2^+ Oxaliplatin 60 mg)[4]. The CRT completion rate was 92.5% (75/81 patients). Six patients stopped treatment due to gastrointestinal or cutaneous toxicity and, among them, 3 patients completed only the first three weeks of treatment. In all cases surgery was planned and performed 6–8 weeks after the end of CRT. The study was submitted to and approved by the Ethic Institutional Review Board for "Biobanking and use of human tissues for experimental studies" of the Pathology Service of the Azienda Ospedaliera Città della Salute e della Scienza di Torino, Torino, Italy. The project provided a verbal and not written informed consent from the patients due to the retrospective approach of the study, which did not impact on their treatment. All the cases were anonymously recorded. The Institutional Review Board approved this consent procedure.

### Histopathological evaluation

All hematoxylin and eosin (H&E)-stained slides available from biopsies and surgical specimens were reviewed, and a representative paraffin block was selected for each case. Biopsy and surgical specimens of each patient were independently reviewed by two dedicated pathologists (RS and PC), who confirmed all diagnoses. On the rectal pre-treatment biopsies, the tumor grade (G1-G2-G3) and apoptosis were evaluated. Apoptosis was assessed by evaluating the apoptotic bodies lining the basal membrane, and it was scored as negative (<25% per 100 viable cells) or positive (> = 25% per 100 viable cells). Vascular invasion was not included among the histopathological parameters analyzed since biopsy samples are usually superficial and small-sized and therefore considered poorly reliable for the presence of neoplastic emboli. Response to neoadjuvant CRT was established by a histopathological examination of surgically resected rectal specimens using the tumor regression grade (TRG), according to Mandard criteria [43], which are classified into five grades from TRG1 (complete regression) to TRG5 (no regression) based on the presence of residual cancer cells and on the degree of fibrotic changes. To simplify the analysis, complete tumor response (TRG1) was compared with partial/absent response (TRG2-5).

### Immunohistochemistry

Immunohistochemistry was performed in all but five cases of pre-treatment biopsies, which had insufficient tissue from the rectal biopsy. Resected surgical specimens with residual cancer (TRG2-5) were analyzed as well. Three ìm thick serial paraffin sections of each case were processed by immunohistochemistry using an automated immunostainer (Ventana BenchMark AutoStainer, Ventana Medical Systems, Tucson, AZ, USA) with antibodies against YKL-40 (Quidel Corporation, San Diego, CA, USA, rabbit polyclonal, diluted 1/400) and c-Met (Abcam PLC, Cambridge, UK, rabbit polyclonal, diluted 1:200). The antigen retriewal step was included in the automated programme. A biotin-free, dextran chain-based detection system (EnVysion, Dako, Carpinteria, CA, USA) and diaminobenzidine (Ventana Medical Systems, Tucson, AZ, USA) were used as the chromogen, according to standard protocols. Neoplastic tissues were assembled on tissue microarrays, excluding the primary antibody and IgG-matched serum, and were used as positive and negative controls.

### Staining interpretation and scoring system

All immunostained slides were analyzed independently by RS, AC and PC, who were blinded to the clinical data. YKL-40 and c-Met staining was assessed as both categorical (negative or positive if present in at least 1% of the neoplastic cells) and continuous variables (scored as number of positive neoplastic cells). When positive, cells had a moderate to strong cytoplasmic reactivity for both antibodies. In case of discrepancies, slides were reviewed using a multihead microscope, and a consensus was reached.

### c-Met Fluorescent in situ hybridization (FISH) analysis

c-Met FISH analysis was performed using the probe mixtures set, MET/CEP7 (Abbott Molecular, Des Plaines, IL, USA), in all biopsies with a positive c-Met immunostaining. FISH experiments were carried out according to the manufacturer’s instructions as follows: 10 μl of probe mixture was applied to each slide, which was immediately covered with a coverslip and sealed with rubber cement. Slides were thoroughly co-denatured in the Hybrite System (Vysis, Downers Grove, IL, USA) at 73°C for 3 minutes and hybridized overnight at 37°C. A post-hybridization wash was performed in 2xSSC-0.3% NP-40 at 73°C for 3 minutes. Then, slides were dehydrated and counterstained with DAPI I (4,6-diamidine-2-phenylindol, Vysis, Downers Grove, IL, USA). An automated scanning station, MethaSystems (Carl Zeiss MetaSystems Gbmh, Altlussheim, Germany), equipped with an AxioImager–Z1 epifluorescence microscope was used to determine the c-Met gene status. Tumor areas with high signal quality and good nuclei preservation were assessed. These areas were automatically scanned, and 10 different consecutive focal planes were made for each FISH signal to form a single bidimensional image. For traditional reading, the automatically acquired images were transferred to the Isis software (Carl Zeiss MetaSystems Gbmh, Altlussheim, Germany) and stored in dedicated files. FISH data were analyzed using previously described criteria, according to Capuzzo et *al*. [44].

### Statistical Analysis

Correlations between YKL-40 and c-Met antibodies were made using the Chi-square test and two-tailed Spearman’s test when considered categorical or continuous variables, respectively. Clinico-pathological parameters for univariate analyses to determine predictors of the response to CRT included tumor grade, apoptosis, YKL-40 and c-Met staining patterns. Fisher’s exact or Chi-square tests were used. All parameters in the univariate analysis with a significant impact on predicting CRT response were considered for multivariate analysis using the Cox proportional hazard model. To analyze the prognostic impact of all pathological variables considered, the univariate overall survival analysis was based on the Kaplan–Meier product limit estimate of survival distribution. Unadjusted differences between survival curves were tested using the log-rank test. All tests were performed using the Statistical Package for the Social Sciences version 17 (SPSS Inc., Chicago, IL, USA). A significance level of p<0.05 was used.

## Results

### 1) Clinicopathological data and pathological tumor response evaluated according to Mandard TRG

The main clinical and pathological features of the whole series of 81 cases are summarized in **Table 1**.

**Table 1:** Clinical pathological features of the 81 rectal cancer biopsies cases analyzed for YKL-40 and c-Met.

Parameter	
**M/F ratio**	1.79
**Age, mean (years) [range]**	63 [44–91]
**Grade**	1: 4
	2: 66
	3: 11
**Mandard TRG**	1: 20
	2: 16
	3: 22
	4: 21
	5: 2
**Lymph node status (pN)**	0: 59
	1: 21
**Disease status** *(lost to FU*: *3)*	NED: 59
	AWD: 18
	DOD: 3
**Local recurrence [median months]**	11 [16]
**Median overall survival (months) [range]**	40 [6–79]
**Median disease free survival (months) [range]**	31 [5–78]

***Abbreviations*:** F, female; M, male; TRG, tumor regression grade; FU, follow up; NED, no evidence of disease; AWD, alive with disease; DOD, died of disease.

**Fig 1:**
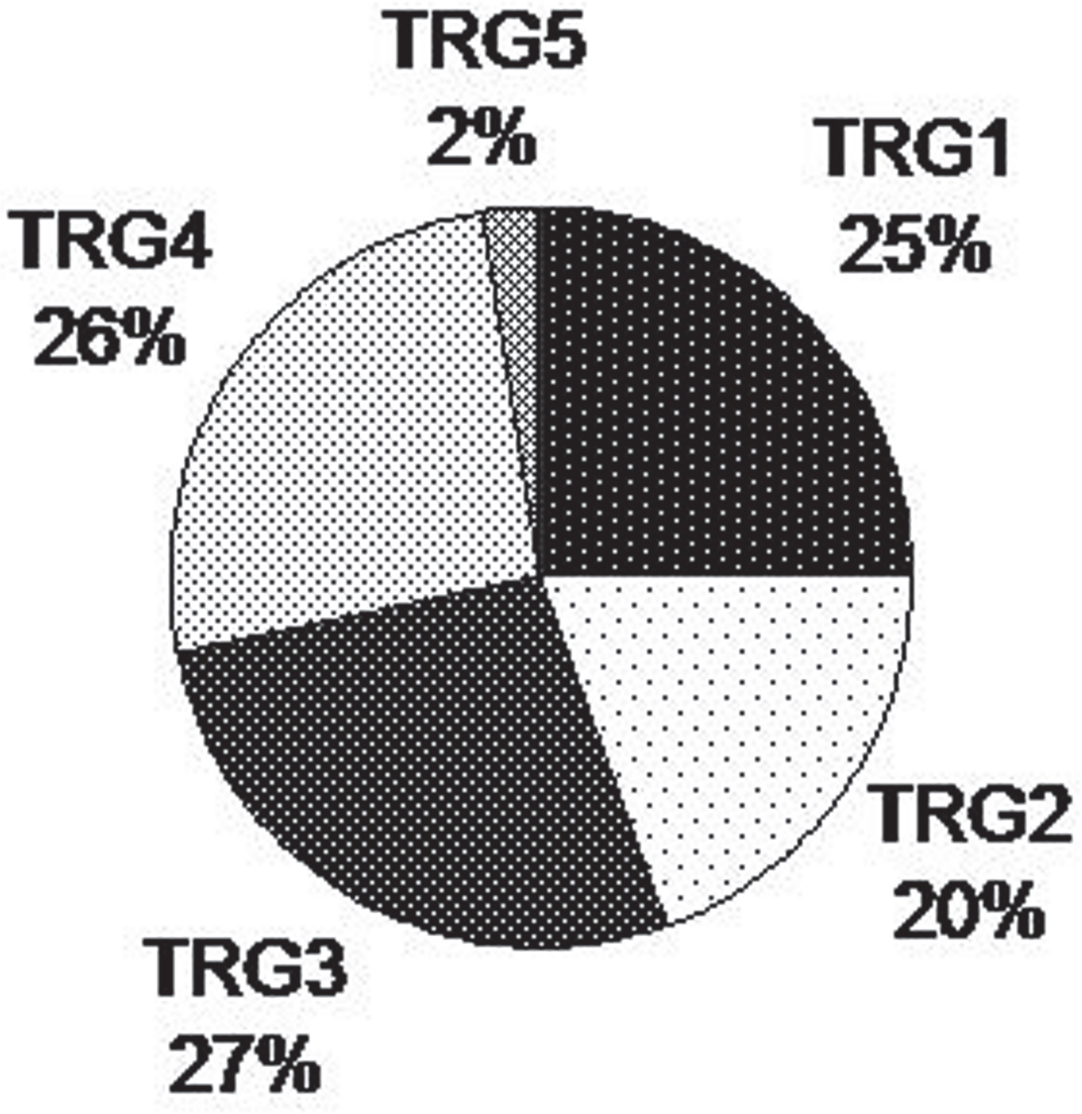
Distribution of patients according to TRG classes.

According to TNM staging, 20 cases (25%) had a post-CRT pathological T stage ypT0, 4 cases (5%) had ypT1, 20 cases had (25%) ypT2, 35 cases had (43%) ypT3 and 2 cases had (2%) ypT4. Lymph node metastases were detected in 22 cases (27%). The clinical T stage (cT) was obtained in 74 cases (91%), and a T-downstaging after RCT was observed in 48 (65%) of them. Applying the Mandard criteria to define the TRG, 20 cases (25%) had a complete pathological tumor response to neoadjuvant CRT (TRG1-ypT0) on the surgically resected rectal specimens. In the remaining 61 patients (75%), a partial or absent tumor response was observed, with 16 cases (20%) classified as TRG2, 22 cases (27%) classified as TRG3, 21 cases (26%) classified as TRG4 and 2 cases (2%) classified as TRG5 (**Fig 1**).

The distribution of complete (TRG1) *versus* partial responders (TRG2-5) was similar in the two subgroups of treatment (RT+capecitabine or XELOXART protocol).

### 2) Immunohistochemistry for YKL-40 and c-Met, FISH analyses and clinicopathological correlates

**Fig 2:**
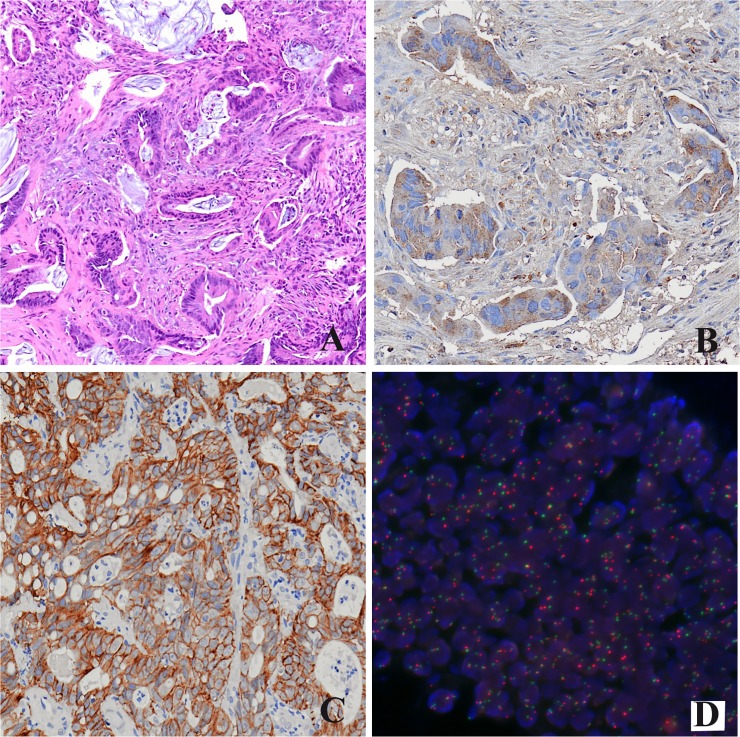
Representative examples of rectal carcinoma (A, Hematoxylin & Eosin, original magnification 200X) with mild to strong cytoplasmic immunoreactivity for YKL-40 (B, original magnification 400X) and c-Met (C, original magnification 400X) and high c-Met gene polisomy by FISH analysis.

Overall, YKL-40 tumor expression was observed in 47 (62%) cases, with a moderate or strong immunostaining localized within the cytoplasm of neoplastic cells (**Fig 2A and 2B**), while forty-three (56%) cases were positive for c-Met with a moderate or strong cytoplasmic reactivity (**Fig 2A and 2C**).

Both YKL-40 and c-Met tumor expression was retained in resected rectal specimens according to their expression on endoscopic biopsies. c-Met FISH analysis was performed in all 43 c-Met-positive cases. No c-Met gene amplification was found, whereas nine (21%) cases demonstrated low/high gene polisomy (**Fig 2D**). The expression of YKL-40 and c-Met was significantly correlated when considered both continuous variables (r = 0.43 and p = 0.001) and categorical variables (χ^2^ = 6.96; p = 0.01). YKL-40 was positive in moderately or poorly differentiated cases (p = 0.018) and in those lacking inflammatory infiltrate (p = 0.01), whereas c-Met was positively correlated with the absence of apoptosis (**Table 2**).

**Table 2:** YKL-40 and c-Met immunohistochemistry: clinical and pathological correlates.

Parameter		YKL-40 negative	YKL-40 positive	*P*	c-Metnegative	c-Met positive	*P*
**Sex**	M	18	29	0.974	13	27	0.619
	F	11	18		10	16	
**Age**	mean	61	64	0.31	65	63	0.404
**Tumor differentiation**	G1	3	0	***0*.*018***	1	0	0.229
	G2	20	43		18	39	
	G3	6	4		4	4	
**Tumor invasion depth**	pT0	12	6	0.06	10	6	0.106
	pT1	1	3		1	3	
	pT2	5	13		4	12	
	pT3	11	23		7	21	
	pT4	0	2		1	1	
**Lymph node metastasis**	N0	23	31	0.299	17	32	0.964
	N1	6	16		6	11	
**TRG**	1	12	6	***0*.*006***	10	6	***0*.*01***
	2–5	17	41		6	37	
**Apoptosis**	absent	11	25	0.196	5	24	***0*.*008***
	present	18	22		18	19	

***Abbreviations*:** F, female; M, male; TRG, tumor regression grade.

### 3) Correlation of Mandard TRG with morphological, immunohistochemical and molecular features (Table 3)

**Table 3:** Univariate analysis for CRT response in 81 patients with locally advanced rectal cancer.

Parameter		TRG1	TRG2-5	*P*
**Sex**	M	11	41	0.323
	F	9	20	
**Age**	mean	61	63	0.347
**Tumor differentiation**	G1	1	3	0.624
	G2	15	51	
	G3	4	7	
**Lymph node metastasis**	N1	18	41	0.08
	N2	2	20	
**Apoptosis**	absent	7	32	0.205
	present	13	29	
**YKL-40** *	positive	12	17	***0*.*006***
	negative	6	41	
**c-Met** *	positive	10	13	***0*.*01***
	negative	6	37	

*: parameters included in the **multivariate analysis**: positive YKL-40: p = 0.004; positive c-Met: p = 0.007.

***Abbreviations*:** F, female; M, male; TRG, tumor regression grade.

**Fig 3:**
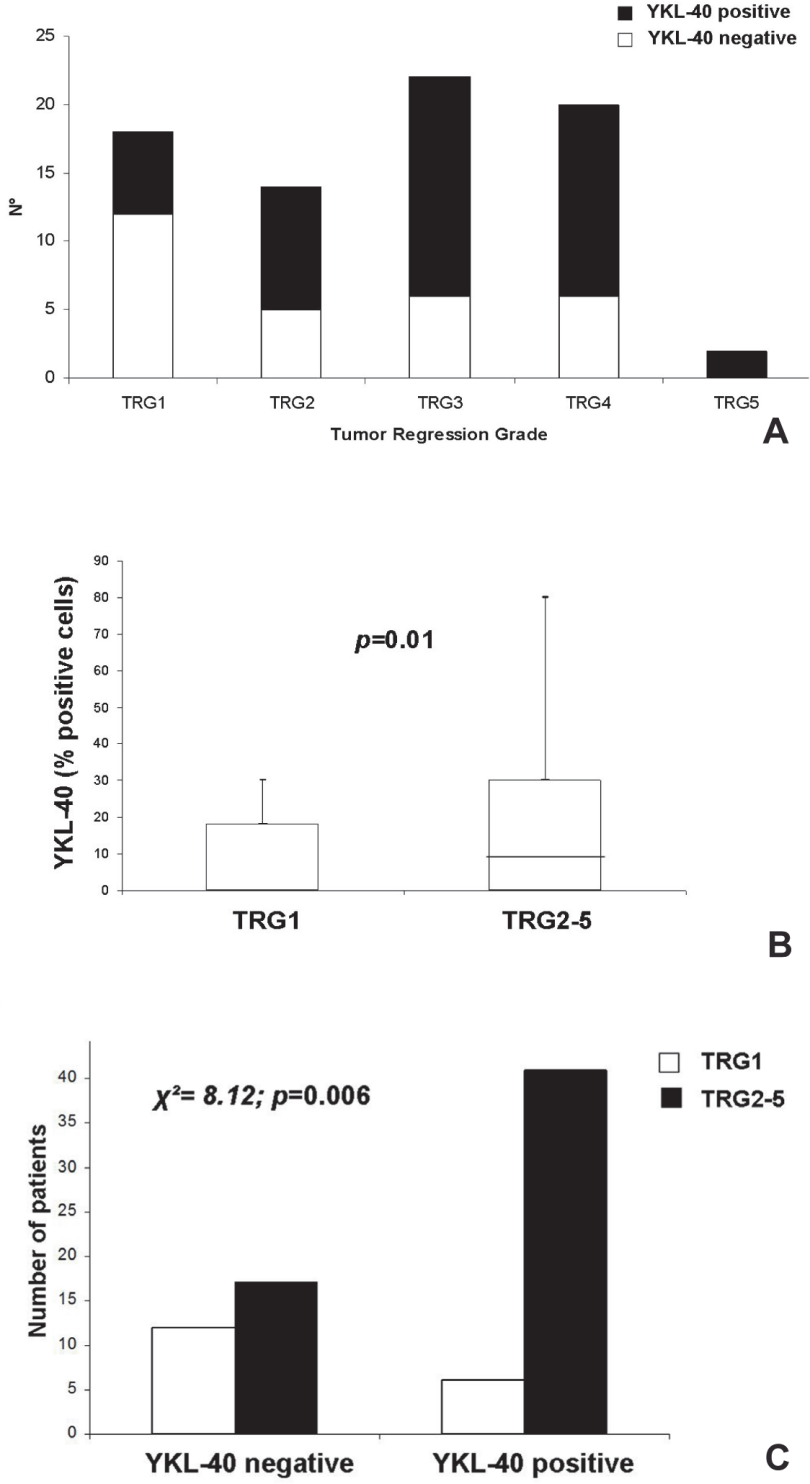
Distribution of cases into to TRG classes according to YKL-40 immunoreactivity (A) and overexpression of YKL-40 in the non-responder group (TRG2-5) when considered both as continuous (B) and categorical variable (C).

**Fig 4:**
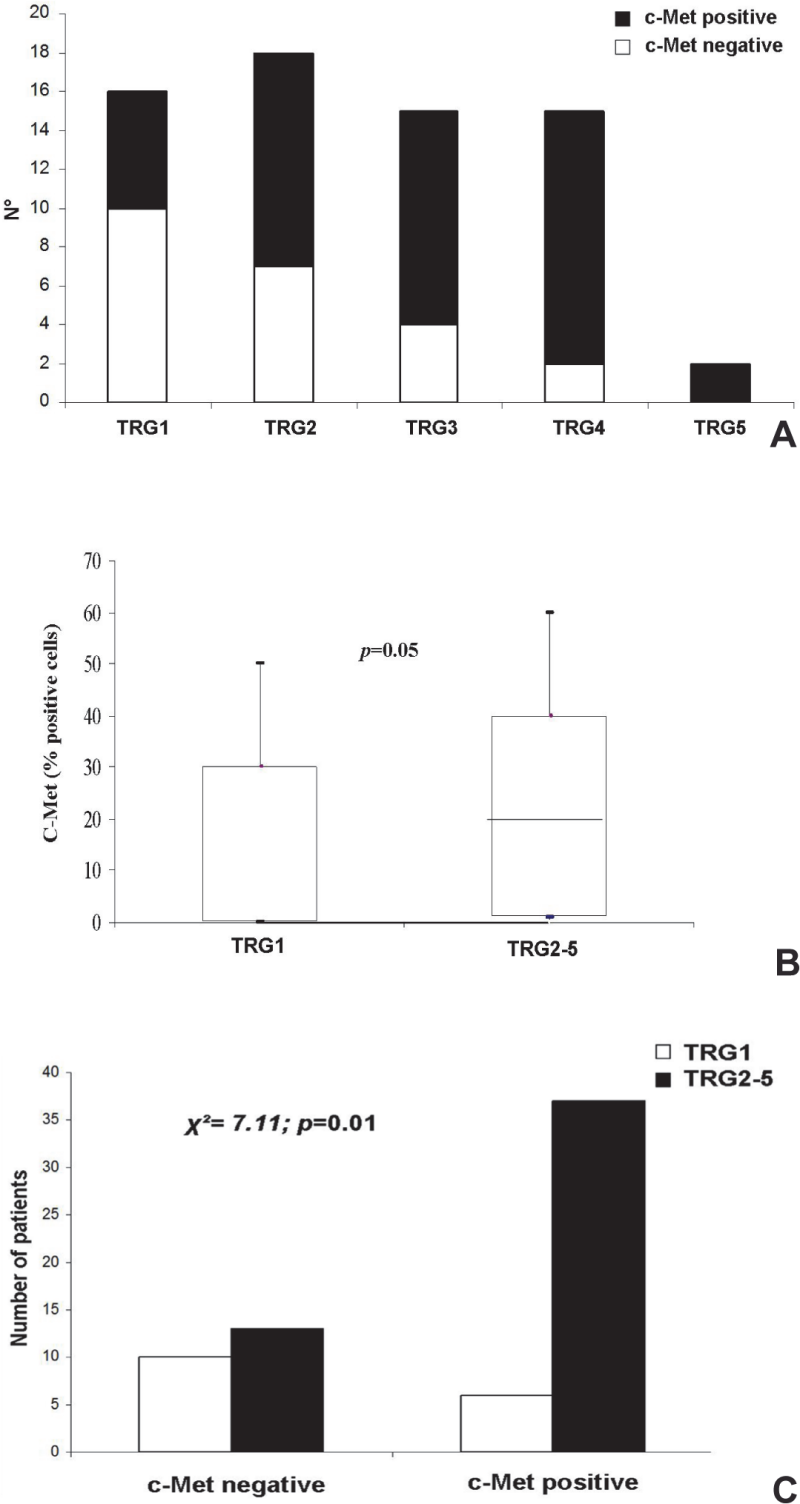
Distribution of cases into to TRG classes according to c-Met immunoreactivity (A) and overexpression of c-Met in the non-responder group (TRG2-5) when considered both as continuous (B) and categorical variable (C).

In a univariate analysis subdividing the entire series into complete (TRG1) or non-complete responders (TRG2-5), neither tumor grade nor the presence of apoptosis were detected on rectal pre-treatment biopsies with TRG, indicating that none of these histological features proved effective in predicting CRT response. Conversely, YKL-40 and c-Met were differentially expressed into the five TRG subclasses (**Figs 3A** and **4A**).

In the TRG1 subgroup, the YKL-40 tumor expression ranged from 0 to 30% (mean: 7.7; median: 0), and that of c-Met ranged from 0 to 50% (mean: 12.5; median: 0); specifically YKL-40 and c-Met immunoreaction was absent in 67% and 62% of TRG1 cases, respectively. In the group of partial responders (TRG2-5), the YKL-40 expression varied from 0 to 80% (mean: 17.65; median: 10), and that of c-Met ranged from 0 to 60% (mean: 23.3; median: 20). Even when considered dichotomized variables, the expression of both biomarkers was significantly associated with a lack of complete tumor regression: 87% of YKL-40-positive cases and 86% c-Met-positive cases had a TRG2-5. The overexpression of YKL-40 and c-Met in the non-responder group (TRG2-5) compared with the responder group (TRG1) was statistically significant, when considered both continuous (p = 0.01 and p = 0.05 or YKL-40 and c-Met expression, respectively, **Figs 3B and 4B**) and categorical variables (p = 0.006 and p = 0.01, respectively) (**Figs 3C and 4C**), and it had high positive predictive values (PPV = 87% and 86% for YKL40 and c-Met, respectively) (**Table 4**).

**Table 4:** Predictive value of YKL-40 and c-Met as marker of chemo-radioresistance.

	YKL-40		c-Met	
	TRG1	TRG2-5	TRG1	TRG2-5
**Sensitivity**		71%		74%
**Specificity**	66%		62%	
**Positive predictive value**		87%		86%
**Negative predictive value**	41%		43%	
**Accuracy**		70%		71%

**Fig 5:**
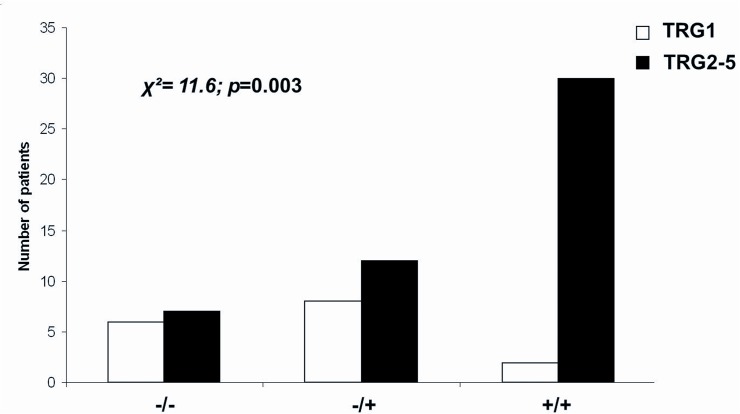
Stratification of cases into three subgroups, according to the expression of YKL-40 and c-Met on tumor biopsy:-/-: YKL-40-negative/c-Met-negative;-/+: YKL-40-positive/c-Met-negative or YKL-40-negative/c-Met-positive and +/+: YKL-40-positive/c-Met-positive.

To verify whether YKL-40 and c-Met co-expression could correlate with a partial or lack of response to neoadjuvant treatment, all cases were stratified into three subgroups, according to the expression of YKL-40 and c-Met on tumor biopsy: i: YKL-40-negative/c-Met-negative (13/65 cases, 20%), ii: YKL-40-positive/c-Met-negative or YKL-40-negative/c-Met-positive (20/65 cases, 30%) and iii: YKL-40-positive/c-Met-positive (32/65 cases, 50%) (**Fig 5**).

Interestingly, 30 of the 32 rectal biopsies co-expressing both biomarkers were TRG2-5 on surgical specimens (partial/absent response). This finding strengthens their positive predictive value (94%).

When cases were stratified as TRG1-2 vs. TRG3-5 the two biomarkers retained their predictive value, although lower. In detail, 68% of YKL-40-positive cases (p = 0.03) and 65% of c-Met-positive cases (p = 0.01) had a TRG3-5. Stratifying by TRG3, only c-Met retained its value (p = 0.03).

Multivariate analysis confirmed YKL-40 and c-Met as independent predictive factors of poor response to CRT (p = 0.004 and p = 0.007 for YKL-40 and c-Met, respectively).

### 4) Correlation of YKL-40 and c-Met expression with disease outcome

Follow-up data, ranging from 6 to 79 months (median: 40), were available for all but three patients. At the time of analysis, 75 (96%) of the patients were alive, but the remaining three (5%) had died. Overall, recurrent disease after neoadjuvant CRT and surgery occurred in 21 patients (28%) (**Table 1**). In addition, 11 patients (14%) experienced a local recurrence with a median follow-up of 16 months.

All 20 patients identified as complete responders (TRG1) were alive at the time of last follow-up (mean follow-up 52 months), as were 15 of the 16 patients classified as TRG2 (mean follow-up 33 months); one patient in the TRG2 group was lost. The three deceased patients had incomplete tumor response upon surgical rectal specimen (TRG 3, 4 and 5, respectively), followed by tumor recurrence. Univariate survival analysis showed that high TRG was the only parameter significantly correlated with a shorter overall survival (p = 0.01) and disease-free survival (p = 0.004).

## Discussion

In this study, we found that YKL-40 and c-Met immunohistochemical expression in pre-treatment rectal cancer biopsies reliably predicts a partial or lack of response to neoadjuvant CRT in patients with locally advanced rectal adenocarcinoma. Moreover, we demonstrated that c-Met and YKL-40 are independent predictors of poor response to CRT and that Mandard TRG is the only prognostic factor of poor survival.

### Predictive role of YKL-40 and c-Met

To date, neoadjuvant CRT is the standard of care for patients with locally advanced (stages II and III) rectal adenocarcinoma. However, its efficacy is quite unpredictable, with a complete pathological response (ypT0) in up to 30% of cases[25,45,46]. Such a heterogeneous individual response in patients with the same pT stage suggests a pivotal role of intrinsic biological properties of neoplastic lesions in determining CRT susceptibility and highlights the need for a clinicopathological and molecular predictive stratification[25]. c-Met proto-oncogene codifies the kinase tyrosine receptor hepatocyte growth factor (HGFR). Its genetic alterations have been identified in a wide variety of solid cancers, and its expression is increased in metastatic lesions[47] and in cancers of unknown primary origin[48]. As an oncogene, c-Met has a dual effect on cancer cells[49]. On the one hand, it is a master driver of the long-term maintenance of transformed neoplastic phenotype, making cancer cells dependent on its signaling for their growth and survival. On the other hand, it acts as an expedient for cancer survival and dissemination, due to its overexpression in adverse microenvironmental conditions, such as hypoxia[50] and therapeutic ionizing radiations[30,37,40,51]. In this regard, several authors demonstrated an improvement in cellular sensitivity to radiation with c-Met inhibitors and siRNA[30,37,52,53]. YKL-40, also termed chitinase-3-like-1, is a secreted glycoprotein that regulates multiple cellular functions, including cell proliferation and differentiation, protection against apoptosis, stimulation of angiogenesis, inflammation and extracellular tissue remodeling. High plasma levels of YKL-40 were found in a wide spectrum of chronic inflammatory diseases and malignancies[54]. It is expressed by both tumor cells and their surrounding tumor-infiltrating macrophages, stimulating the production of various tumor growth factors, including angiogenic factors, cytokines, and chemokines. Although the overall pathological role and molecular mechanisms of YKL-40 in tumorigenesis remains to be established, its angiogenic signature has been reported to regulate tumor development in breast cancer, colon cancer and glioblastoma [54,55]. Interestingly, ionizing radiations were shown to increase YKL-40 expression and angiogenesis in a glioblastoma cell line, and this condition was reversed by a neutralizing YKL-40 antibody[31]. Moreover, ionizing radiation and neutralizing YKL-40 antibody synergistically inhibited tumor growth in xenografted brain tumor models[34]. The aim of this multicentric study was to verify the usefulness of c-Met and YKL-40 expression in identifying those patients who will scarcely/not benefit from neoadjuvant CRT. c-Met and YKL-40 expression in biopsy specimens had a highly positive predictive value, as they were associated with lack of complete tumor response in 86% and 87% of cases, respectively *(*p = 0.01 and p = 0.006). Interestingly, this observation was further reinforced when only co-expression was considered: 94% of the rectal biopsies co-expressing both biomarkers showed TRG2-5.

Stratifying patients according to TRG2, both YKL-40 and c-Met retained their predictive value (p = 0.03 and p = 0.01 for YKL-40 and c-Met respectively), whereas stratifying by TRG3 c-Met only proved to predict therapy efficacy (p = 0.03).

Such a result is in line with the ‘stress-response’ role of the two molecules: tumors highly expressing c-Met and YKL-40 will most likely not benefit from CRT because these cancer cells have already switched on a transcriptional adaptation to unfavorable microenviromental conditions. The decision to use an immunohistochemical approach instead of a molecular analysis provides practical advantages. Genomic and proteomic analyses are expensive, and special equipment and highly trained technicians are required to perform certain molecular analyses, whereas immunohistochemistry is widely employed in diagnostic routines and has a favorable cost-benefit balance. Based on the previous external validation of these multi-institutional results, the introduction of an immunohistochemistry-based panel composed of YKL-40/c-Met in rectal cancer diagnostic-routine in parallel to H&E-stained slides could be used to avoid excessive costs even in laboratories managing a high number of surgical specimens.

### Prognostic role of clinical and pathological variables

The role of c-Met in prognosis has been reported to correlate with its overexpression in different solid tumors (such as gastric adenocarcinoma[51], ovarian[56] and non-small lung cancer[57]; the overexpression of YKL-40, particularly as a circulating marker[54], is similarly correlated with its role in prognosis. Nevertheless, our results do not support the reported prognostic role for c-Met and YKL-40[58] in colorectal cancer[32,39,41,59], although the latter was described only at the serum level. We found that TRG, quantified in five grades according to the Mandard scale[43], was the sole parameter influencing patient outcome in terms of both overall survival and disease-free survival, as previously reported[60–62]. Accordingly, patients classified as TRG1 (complete tumor response) and TRG2 (single cells or small groups of cancer cells) showed a better overall survival (100% at the time of last follow up in both groups) and control of disease progression (84,2% in TRG1 group and 80% in TRG2 group). Although the prognostic and predictive significance of TRG is still influenced by the limited end points and the quality and heterogeneity of histological data, TRG following preoperative chemoradiotherapy for rectal cancer has a solid prognostic evidence. A future challenge remains to discover a surrogate noninvasive method to surgery to accurately identify patients who have obtained a favorable TRG and to use TRG to select patients for postoperative adjuvant chemotherapy following preoperative CRT[63].

## Conclusion

YKL-40 and c-Met expression in pre-neoadjuvant rectal cancer biopsy is a reliable tool to predict the partial or lack of tumor response to CRT. Accordingly, an “immunohistochemistry-enriched” diagnostic approach to rectal biopsies, aimed at increasing CRT sensitivity using specifically selected targeted therapies for those expected to be poor responders, could provide predictive information with cost-benefit efficacy and facilitate the further therapeutic selection of patients.

Finally, prospective randomized trials are necessary to validate the role of YKL-40/c-Met in predicting tumor resistance; if so, the efficacy of neoadjuvant CRT in these patients would benefit of therapy sensitizers, i.e. targeted c-met inhibitors, to improve responsiveness.
